# Rapid analysis of intraperitoneally administered morphine in mouse plasma and brain by microchip electrophoresis-electrochemical detection

**DOI:** 10.1038/s41598-019-40116-5

**Published:** 2019-03-01

**Authors:** Elisa Ollikainen, Teemu Aitta-aho, Michaela Koburg, Risto Kostiainen, Tiina Sikanen

**Affiliations:** 1 Drug Research Program, Division of Pharmaceutical Chemistry and Technology, Faculty of Pharmacy, University of Helsinki, Helsinki, Finland; 2 Department of Pharmacology, Faculty of Medicine, University of Helsinki, Helsinki, Finland

## Abstract

Animal studies remain an essential part of drug discovery since *in vitro* models are not capable of describing the complete living organism. We developed and qualified a microchip electrophoresis-electrochemical detection (MCE-EC) method for rapid analysis of morphine in mouse plasma using a commercial MCE-EC device. Following liquid-liquid extraction (LLE), we achieved within-run precision of 3.7 and 4.5% (coefficient of variation, CV, n = 6) and accuracy of 106.9% and 100.7% at biologically relevant morphine concentrations of 5 and 20 µM in plasma, respectively. The same method was further challenged by morphine detection in mouse brain homogenates with equally good within-run precision (7.8% CV, n = 5) at 1 µM concentration. The qualified method was applied to analyze a set of plasma and brain homogenate samples derived from a behavioral animal study. After intraperitoneal administration of 20 mg/kg morphine hydrochloride, the detected morphine concentrations in plasma were between 6.7 and 17 µM. As expected, the morphine concentrations in the brain were significantly lower, *ca*. 80–125 nM (280–410 pg morphine/mg dissected brain), and could only be detected after preconcentration achieved during LLE. In all, the microchip-based separation system is proven feasible for rapid analysis of morphine to provide supplementary chemical information to behavioral animal studies.

## Introduction

Morphine, a narcotic drug used to treat severe pain, has a long history as a postoperative analgesic in clinical settings. However, there is notable individual variability in response to morphine, ranging from analgesia to euphoria, which makes morphine one of the most widely studied neuroactive compounds in pharmacological settings^1^. The variability of response is often associated with morphine metabolism, but results from human studies are not unanimous^1^ and much relies on preclinical animal studies. In addition to monitoring of the behavioral changes between animals, chemical analysis of endogenous and exogenous compounds in animal-derived samples provides valuable supplementary information to understanding the pathogenesis of, for instance, nociception, analgesia, and addiction. However, conventional chemical analysis techniques are often time-consuming and require large and expensive instrumentation, which are not easily accessible in animal laboratories nor in clinical settings. The detection of morphine typically rests upon pretreatment by solid-phase extraction (SPE) followed by liquid chromatographic (LC) separation coupled with a variety of detection techniques ranging from fluorescence^2^ to electrochemistry (EC)^3–5^ and mass spectrometry (MS)^6–9^. While LC-MS and LC-EC are well-established techniques, there is a constant need for faster and simplified approaches that preferably enable instant analysis of animal samples and data interpretation alongside behavioral monitoring.

Miniaturization of the analytical systems, particularly capillary electrophoresis devices, enables fast and low-cost analysis with very low sample and solvent consumption^10,11^. The possibility to build up hand-held instrumentation further increases the appeal to use miniaturized separation systems for on-site analyses, not only in animal laboratories, but also in the clinic or the environment. With a view to morphine detection, miniaturized capillary electrophoresis devices are also coupled with a variety of detectors^12^, but as the sample volumes are typically very small, the sensitivity is an issue and requires capability to detect only attomoles of chemicals^13^. The use of optical spectroscopic techniques is therefore often compromised by the decreased optical path length, which impairs the detection sensitivity^11,13^. Miniaturization of electrospray ionization (ESI) provides increased sensitivity in MS detection, but realization of the on-chip ESI emitters often requires special cleanroom processes^14^. Mass spectrometers are also expensive and need special laboratory facilities and educated operator, which does not favor the use of MS in an animal laboratory. Instead, implementation of the EC electrodes *via* thin-film metallization is a well-established microfabrication process^15^, and the sensitivity of EC detection does not suffer from miniaturization. As a result, EC detection remains as the most feasible method for routine analysis of morphine from animal samples in a pharmacological setting. Before this work, microchip electrophoresis-electrochemical detection (MCE-EC) has already proven to be a viable method in the analysis of, *e.g*., cellular nitrosative stress markers^16^ and a range of electroactive standards, including neurotransmitters, vitamins, flavones, and water pollutants^15,17–19^. MCE-EC has also been used to quantify hydrogen peroxide^20^ and primary amines^21^ in mouse microdialysis samples. Zhang *et al*. demonstrated MCE-EC analysis of morphine and codeine in human urine after oral administration of cough syrup^22^. Although the feasibility of MCE-EC for a range of applications has been shown, and commercial instrumentations also exist, there is still a gap between the early-adopters (lab-on-a-chip community) and the envisioned end-users (*e.g*., pharmacologists) of the MCE-EC technology. In the absence of comprehensive validation (sensitivity, robustness) with real-scale sample sets, adaptation of the technology to routine analysis appears risky and uncertain. The purpose of this study is to bridge the gap in implementation of the MCE-EC methodology in animal laboratories by demonstrating its feasibility for the targeted analytical need of morphine detection in mouse plasma and brain. To demonstrate the power of the commercial microchip-based separation system, we developed and qualified an MCE-EC method with mouse plasma and brain homogenate samples derived from a pharmacological setting which monitored the behavioral effects of morphine in mice. Instead of the commonly used SPE-based pretreatment, a liquid-liquid extraction (LLE) method was adopted for rapid sample preparation prior to MCE-EC analysis, because it is instrument-free and easily scaled down to low sample volumes.

## Results and Discussion

### Optimization of the separation method

**Figure 1:**
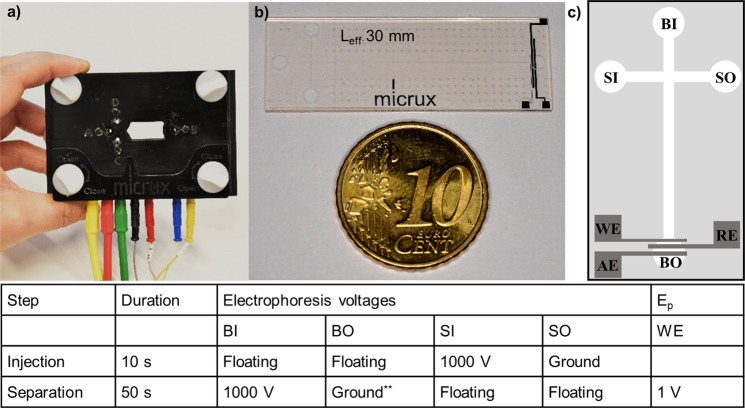
Photographs of (**a**) the chip holder and (**b**) the glass/SU-8 microchip, and (**c**) a schematic view of the microchip design (not in scale). The microchip consists of a 35-mm-long separation channel (50 µm × 20 µm, w × h) intersected by a 10-mm-long injection channel (50 µm × 20 µm, w × h; simple cross). The effective separation length (L_eff_) between the injection cross and BO is 30 mm. (BI) buffer inlet; (BO) buffer outlet; (SI) sample inlet; (SO) sample outlet; (WE) working electrode; (RE) reference electrode; (AE) auxiliary electrode. The optimized electrophoresis and EC detection voltages during injection and separation steps are given below the figure in tabular form. ^**^BO electrode also served as the auxiliary electrode for amperometric detection.

**Figure 2:**
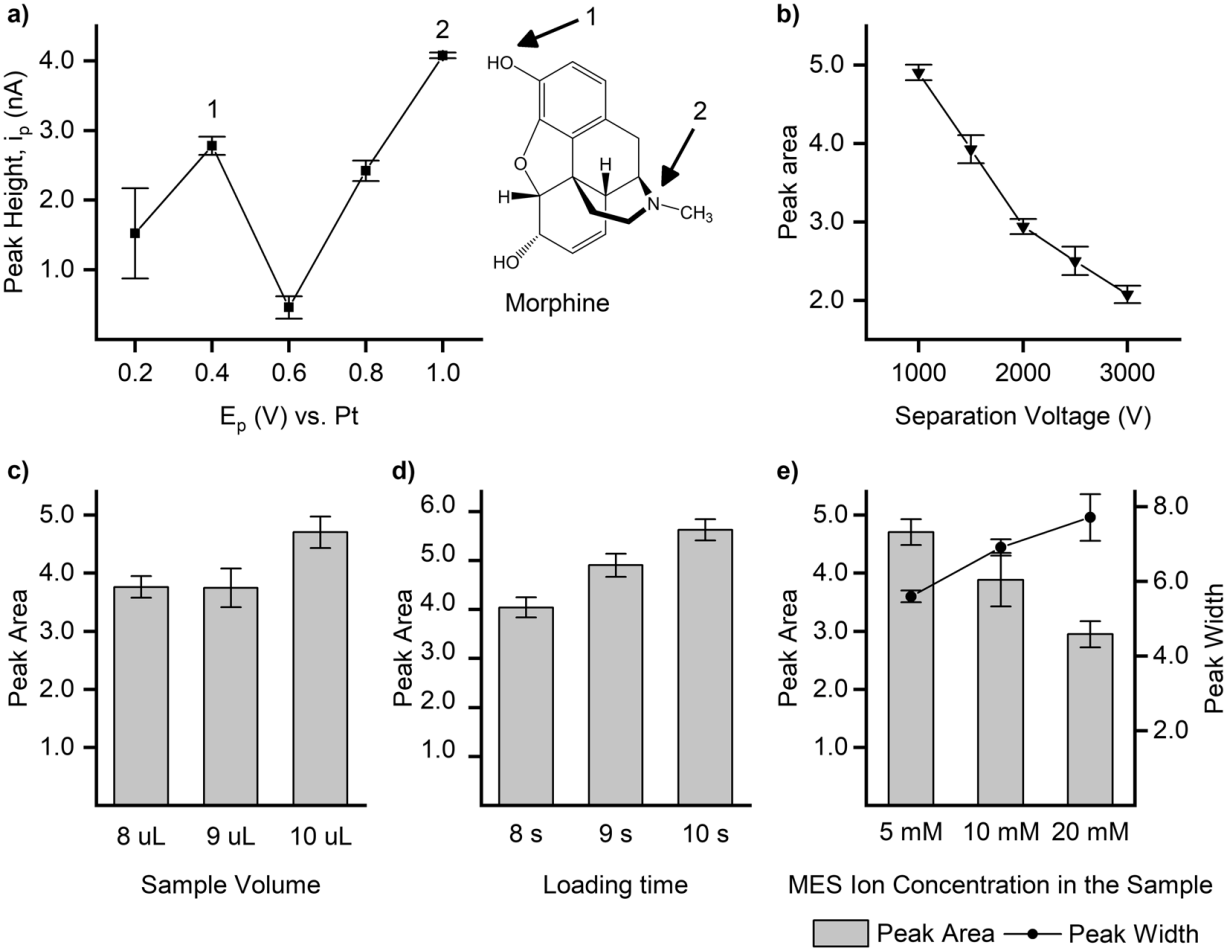
(**a**) Effect of working electrode potential (E_p_) on the peak height (i_p_) (n = 4 repeated runs). Effects of (**b**) separation voltage (n = 4 repeated runs), (**c**) sample volume (applied to the sample inlet, n = 5 repeated runs), and (**d**) sample loading time (n = 5 repeated runs) on the morphine peak area. (**e**) Effect of MES ion concentration in the sample solution on the morphine peak area and peak width (n = 6 repeated runs). The morphine concentration was 50 μM in (**a**,**b**), 20 μM in (**d**), and 10 μM in (**c,e**). The background electrolyte in all analyses was 20 mM MES (pH 6.5) and the working electrode potential (E_p_) was constantly 1.0 V (except in **a**).

A commercial microchip electrophoresis instrument with amperometric detector (MicruX Technologies, Oviedo, Spain) was used in the study (Fig. 1a,b). The system is portable and includes a high voltage power supply for application of the electrophoresis separation voltages, a biopotentiostat for application of the detector voltages, an electrophoresis chip (consumable part), and a chip holder with electrical connectors facilitating application of the separation and detector voltages to the microfluidic chip^17,23^. The microfluidic electrophoresis chip is polymer (SU-8™) based and comprises of sample inlet (SI) and outlet (SO) and buffer inlet (BI) and outlet (BO) interconnected with microfluidic channels (Fig. 1c). In addition, the chip features integrated thin-film metal (titanium/platinum 50/150 nm) working (WE), auxiliary (AE) and reference (RE) electrodes close to the buffer outlet (Fig. 1c). In MCE, sample loading prior to separation is carried out by applying high voltage to SI, while grounding SO, through electrical connectors embedded in the corresponding liquid reservoirs of the chip holder. This gives rise to electroosmotic flow (EOF) in the injection channel (SI→SO), which intersects the separation channel (BI→BO) in its beginning and delivers the sample to the injection cross. During sample loading, the electrodes at BI and BO reservoirs are floating so there is no EOF in the separation channel. Next, sample injection is performed by applying high voltage to BI, while grounding BO and leaving SI and SO floating, which induces EOF in the separation channel (BI→BO) but not in the injection channel (SI→SO). The injected sample volume (here, 50 pL) is defined by the channel dimensions at the injection cross. After injection, the sample components migrate in an electric field applied across the separation channel (filled with background electrolyte, BGE) and are separated from each other based on their size and charge. Over the entire sample loading-separation cycle, constant voltage is applied to the WE, while keeping AE grounded and RE floating. When the sample components reach the integrated thin-film electrodes at the separation channel outlet (Fig. 1c, BO), the components are detected based on change in the detector current (WE→AE) as the result of their characteristic oxidation or reduction reactions induced by the potential (E_p_) applied to the working electrode (WE) through electrical connectors embedded to the chip holder. All sample loading, separation and detector potentials are controlled *via* the software provided together with the commercial instrument. The optimized separation and detector voltages at each step are given in a tabular form in Fig. 1. In this study, the MCE separation and EC detection conditions were optimized in terms of selectivity, sensitivity, and repeatability. MES (2-(*N*-Morpholino)ethanesulfonic acid) buffer (20 mM, pH 6.5) was selected as the BGE since it provided low background (electrical noise) and thus facilitated sensitive detection of morphine. The mechanistic basis for EC detection of morphine is well-established in the literature^24,25^ describing three anodic peaks at positive WE potentials that can be associated with the oxidation of the phenolic group at the 3-position ( + 0.45 V), further oxidation of the dimeric pseudomorphine generated from the previous oxidation product ( + 0.8 V, at basic pH only), and a two-electron oxidation of the tertiary amine group to form a secondary amine ( + 1.0 V). Optimization of the working electrode (WE) potential showed that the peak potential (E_p_) of + 1.0 V provided the best performance considering both within-run precision (0.7% CV, n = 4 runs, 50 µM morphine) and the signal-to-noise ratio of the peak height (Fig. 2a). Last, the separation efficiency and selectivity were optimized by varying the separation voltage between 1000 V (practical lowest limit for maintaining stable electroosmotic flow) and 3000 V (maximum instrument output) with increments of 500 V. The best resolving power between morphine and the background interferences was obtained using separation voltage of 1000 V (333 V/cm), which facilitated straightforward integration of the peak area and thus the best sensitivity (Fig. 2b). The migration time of morphine under these conditions was 22.0 ± 0.8 s with good between-run precision (3.7% CV, n = 6 runs on three different days, 50 μM morphine).

The robustness of the method with a view to morphine quantitation was studied using morphine concentrations of 10 and 20 μM. The effects of sample loading time (8–10 s) and the sample volume (8–10 µL applied to the sample inlet) on the obtained morphine peak areas were compared. In addition to sensitivity (obtained peak area), both loading time and applied sample volume may affect the peak shape and cause baseline drift *via* hydrostatic pressure anomalies^26^, which is typically manifested by poor repeatability. In this study, further optimization of the peak shape was achieved by reducing the ion concentration of the sample buffer from 20 mM (as in BGE) to 5 mM. Owing to the lower ion concentration in the injected sample plug, electrokinetic stacking takes place during the separation and serves as an additional on-chip sample enrichment step. The lower ion concentration (5 mM) of the sample yields lower conductivity and thus higher electric field strength and faster migration of the sample ions within the sample plug compared to BGE (20 mM), and this eventually results in enrichment of cationic morphine (pK_a_ 9.85 at 20 °C)^27^ to the front of the sample plug. As expected, increases in the sample loading time and applied sample volume resulted in somewhat larger peak areas (Fig. 2c,d), same as electrokinetic stacking (Fig. 2e). However, even if the sensitivity was slightly better, the precision of morphine quantitation (peak area) was shown to be less dependent on the sample loading conditions (time, volume) or the ion concentration (Fig. 2c–e). In other words, all tested conditions provided somewhat similar and acceptable within-run precision between 3.8 and 12.9% CV, suggesting that the quantification capability of the method in terms of precision was not too sensitive to sample loading time, applied sample volume, or the stacking effect in these ranges. In practical terms, this means that as long as the sample loading time and volume are kept constant, the method is sufficiently robust for a nonskilled user without particular need for excessive optimization of the sample loading conditions unless extreme sensitivity is required. This aspect is particularly important with a view to instant analyses in animal laboratories. In this work, however, sample loading time of 10 s and sample volume of 10 µL as well as on-chip sample stacking were used in all further experiments, since these provided the largest peak area and thus the best sensitivity for morphine detection. Under these conditions, the within-run (n = 4) and between-run (n = 4 runs on three different days) precisions of peak area (morphine standards in MES buffer) were 4.3/29.4%, 4.3/5.3%, and 0.9/7.9% (CV) for morphine concentrations of 1, 5, and 20 µM, respectively.

### Optimization of the sample preparation method

Although extraction of morphine in plasma and brain (homogenate) is commonly carried out by SPE^7^, LLE was considered a more feasible combination with the MCE-EC method in this study. In LLE, the sample components are distributed between two immiscible liquids (commonly, aqueous and organic solvents) based on their lipophilicity. LLE does not require any specialty equipment and is more easily scaled up or down depending on the available sample volume. The easy scalability was particularly important in this work to be able to use the same sample preparation and separation protocols for both mouse plasma (very limited sample volume of few tens of μL only, but relatively high morphine concentration) and brain homogenates (larger sample volume of few mL, but low morphine concentration). In addition, volumetric sample preconcentration could be performed during LLE as needed, which was proven to be particularly useful in case of brain homogenates. Compared with low-volume SPE systems (*e.g*. the ZipTips^®^)^28^, LLE also benefits from much less manual work and lower cost. The LLE method used was adopted with modifications from a previous study^29^. The recovery of morphine was determined using blank mouse plasma or brain homogenate (both spiked with known concentration of morphine). The obtained morphine peak areas in different matrices were compared to those of morphine standards dissolved in MES buffer (same morphine concentration, no LLE). As expected on the basis of relatively high degree of morphine binding to plasma proteins^30^, the recoveries of morphine in plasma were relatively low, *i.e*., 28.2 ± 3.4%, 24.4 ± 0.9%, and 30.5 ± 1.4% (mean ± standard deviation, n = 6 of each) at spiked morphine concentrations of 1, 5, and 20 µM, respectively (Table 1). However, the within-run precision was good and the low recoveries could be compensated by the two-fold preconcentration achieved during LLE (*via* sample volume reduction from 100 µL original sample volume to 50 µL redissolution volume) so that biologically relevant morphine concentrations could be reliably detected with the developed method. To be able to detect intraperitoneally administered morphine in the brain homogenates, the preconcentration factor was increased to 16-fold (*via* volume reduction from 400 µL sample volume to 25 µL redissolution volume during LLE). This allowed us to enrich morphine in the brain homogenate samples to detectable (low micromolar) level from the initial (low-nanomolar) level in the dissected mouse brain homogenized with 5 mL deionized water. In the absence of plasma proteins, the recovery of morphine in brain homogenate was much better than that in plasma, *i.e*., 69.4 ± 5.4% at 1 µM concentration (n = 5). Both recoveries and preconcentration factors achieved upon LLE were taken into account in calculation of unknown morphine concentrations in animal samples. For plasma samples, the recovery value of 24.4%, corresponding to that obtained at 5 µM morphine concentration, was used.

**Table 1:** Method qualification results for morphine detection in mouse plasma^a^.

Parameter		Result
Within-run precision	1 µM	11.9%
	5 µM	3.6%
	20 µM	4.5%
Accuracy	1 µM	119.2%
	5 µM	106.9%
	20 µM	100.7%
Recovery	1 µM	28.2%
	5 µM	24.4%
	20 µM	30.5%

^a^All samples were prepared by spiking blank plasma with morphine (n = 6 replicates of each) and treated with LLE before MCE-EC analysis. The separation buffer was 20 mM MES (pH 6.5), the separation voltage 1000 V, and the working electrode voltage 1.0 V. The samples (10 µL) were injected electrokinetically (floating injection) for 10.0 s at 1000 V.

### Method qualification

**Figure 3:**
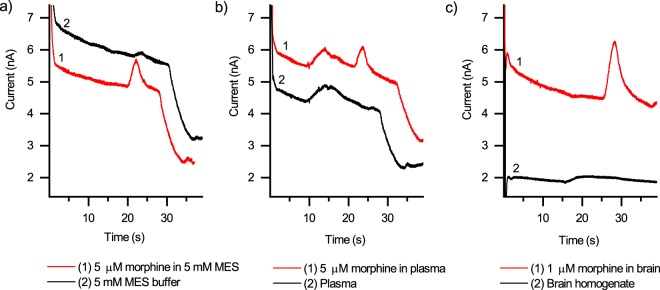
Illustration of the selectivity of analysis of morphine in different matrices compared with blank matrix. (**a**) 5 µM morphine in 5 mM MES buffer, (**b**) 5 µM morphine in mouse plasma, and (**c**) 1 µM morphine in mouse brain homogenate. Plasma and brain samples were treated with LLE before analysis and the residue was dissolved in 5 mM MES (pH 6.0) buffer after evaporating the extraction solvent. All separations were performed at 333 V/cm using 20 mM MES (pH 6.5) as the BGE and working electrode potential of 1.0 V. The loading time was 10 s and the sample volume applied to the SI was 10 µL.

The analytical method was further qualified adopting the U.S. Food and Drug Administration (FDA) guidance^31^ in relevant parts in order to determine selectivity, linearity, within-run precision, accuracy and recovery (of LLE, as described previously). The selectivity of the method was shown to be good and the morphine peak was easily detected and separated from the background interferences originating from buffer, plasma, or brain homogenate (Fig. 3). To be able to reduce the use of matrices of animal origin, linearity was determined with morphine standards diluted in 5 mM MES buffer (pH 6.0), whereas within-run precision, recovery, and accuracy were determined using spiked matrices (*i.e*., plasma or brain homogenate).

The lower limit of quantitation (LLOQ) of the MCE-EC method was preliminarily determined based on precision of morphine quantitation at each concentration in the range of 0.05–0.5 μM. According to the FDA guidance, at LLOQ, CV should not exceed 20% and this was reached at morphine concentration of 0.4 μM (16.4% CV, n = 4). The obtained LLOQ is similar to that previously reported for MCE-EC analysis of morphine in human urine^22^ and corresponds to only 20 amol of morphine per the estimated injected sample volume of 50 pL (calculated on the basis of the simple injection cross dimensions 50 µm × 50 µm × 20 µm, width × length × height). The calibration curve was then established between morphine concentrations of 0.5 and 20 µM, in which range the FDA specifications for within-run precision were fully met (*i.e*., deviation of standards from nominal concentration and precision were less than 15% at all concentrations). By establishing the calibration curve always on the day of use, good within-run precision (between 3.7 and 12.3% CV, n = 6 each) was obtained for morphine detection in plasma at all three concentrations tested (1, 5, and 20 µM). The accuracy of morphine detection in mouse plasma was also within the limits of FDA guidance at 5 and 20 µM concentrations (106.9 and 100.7%, respectively, n = 6 of each concentration), whereas at 1 µM concentration it was 119.2% and thus slightly over the FDA specifications (mean within 15% of the actual value, *i.e*., accuracy between 85 and 115%). However, since the biologically relevant range of morphine concentration in plasma is between 5 and 20 μM^32^, the method was considered feasible for rapid, yet reliable quantitation of morphine in mouse plasma. The method qualification parameters for morphine analysis in plasma are summarized in Table 1.

In addition to plasma samples, the MCE-EC method was challenged by morphine detection in mouse brain homogenates as the sample matrix. *Via* sample preconcentration by LLE (16-fold), the morphine concentration in brain homogenate samples reached detectable levels. The within-run precision and accuracy of morphine detection in preconcentrated brain homogenate (at 1 µM initial concentration) were 7.8% CV and 89.6% (n = 5), respectively, which met the FDA specifications. Other than these parameters, the method qualification parameters for morphine analysis in brain was exactly the same as those used for mouse plasma samples. However, given the uncertainty related to single-point qualification in terms of precision and accuracy, the MCE-EC method was concluded feasible for semi-quantitative detection of morphine in mouse brain to supplement the quantitative data on corresponding plasma concentrations.

### Analysis of intraperitoneally administered morphine in mouse plasma and brain

**Figure 4:**
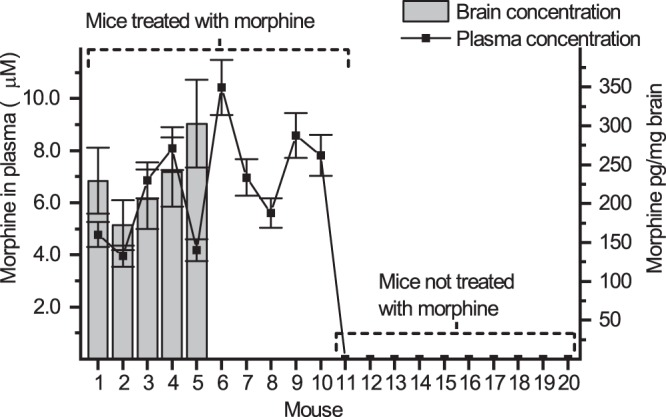
Morphine concentration detected in plasma (scatter graph, left y-axis) and brain (bar graph, right y-axis) for each mouse. The error bars represent propagated error based on precision and recovery.

Determination of morphine in animal-derived samples benefits not only preclinical research but also aids in understanding the pathogenesis of morphine induced side effects, as opioids remain the main choice of pharmacotherapy in major pain indications. To further qualify the method for detection of intraperitoneally administered morphine, mouse plasma and brain homogenate samples derived from a behavioral animal study were utilized to challenge the microchip methodology. Plasma samples collected 30 min after intraperitoneal (*i.p*.) administration of morphine (20 mg/kg) from ten mice and another ten mice not treated with morphine were analyzed with the developed MCE-EC method. The morphine concentrations found in mouse plasma were between 6.7 and 17 µM (Fig. 4), whereas no morphine was detected in plasma samples collected from mice not treated with morphine. The results were very well in line with previously reported morphine concentrations in blood preparations determined using conventional chromatographic separation systems^32^.

In addition, brain homogenates of five of the mice treated with morphine (20 mg/kg, *i.p*) and decapitated 30 min after administration were analyzed. In all cases, intraperitoneally administered morphine reached detectable levels (0.9–1.4 μM) in the samples, which had been preconcentrated by 16-fold during LLE. The back-calculated morphine concentrations (*i.e*. concentrations before preconcentration by LLE) in the mouse brain homogenized with 5 mL of deionized water were between 80–125 nM corresponding to *ca*. 280–410 pg morphine per mg dissected brain. The morphine concentrations detected in brain homogenates followed the same trend as the plasma concentrations in four cases out of five; mice with higher morphine concentration in plasma also had higher morphine concentration in the brain (Fig. 4).

The analytical error, associated with the microchip methodology and the recovery, is expressed with error bars in Fig. 4, and was calculated based on the precision of the MCE-EC method and the LLE by taking into account the average recoveries and preconcentration factors of plasma and brain samples. In addition to analytical error, the success of the morphine dosage to mice may cause variation in morphine concentrations between animals. In all, the results suggest that the developed MCE-EC method is feasible for monitoring the pharmacokinetic differences in plasma concentrations of intraperitoneally administered morphine between mice. Furthermore, the same method may be used for semi-quantitative detection of corresponding alterations in morphine levels in the brain, in case the animal is truncated.

## Conclusions

Despite the well-established feasibility of microchip-based separation systems for a range of bioanalytical applications, the microchip technology is still sparsely applied to routine analysis, *e.g*., in the clinic or animal laboratories. The goal of this work was to bridge the gap in implementation of microchip technology to routine analysis between the early-adopters (lab-on-a-chip community) and the envisioned end-users (*e.g*., pharmacologists) by developing a qualified MCE-EC method for a targeted analytical need, *i.e*., analysis of morphine in mouse plasma and brain, using a commercial MCE-EC platform. In addition to the consumable part (the electrophoresis chips), the commercial setup also includes portable, plug-in control instrumentation (MicruX Technologies), so it is accessible to any average user with reasonably low technical and financial input. Apart from the control instrument, the set-up or the developed method does not require any special equipment or skills. Sample preparation is based on low-cost and equipment-free LLE (off-chip) accompanied by on-chip sample enrichment *via* electrokinetic sample stacking. As a result, the sample preparation can be easily up- and downscaled for different sample volumes on demand (with possibility of simultaneous volume-based sample preconcentration during LLE). Amperometric detection does not require labeling of the sample components prior to detection. Minor variation in the critical sample loading parameters (*i.e*., loading time, applied sample volume), which may easily arise when operated by a nonskilled user, did not significantly affect the critical performance parameters (*e.g*., precision). Accuracy of the method was good with both plasma and brain as the sample matrix despite the fact that method development was partly carried out with help of standard solutions (in order to minimize the unnecessary use of animal-derived matrices). The results suggested that the microchip-based method is feasible for rapid analysis of animal-derived samples with good within-run precision and accuracy, which often confine the adaptation of microchip-based analytical methods to real use. The small and portable instrumentation allows instant analysis at the site of research (such as animal research facilities) and is thus likely to open up new possibilities for implementing chemical information as an integral part of behavioral data derived from animal studies. The possibility to determine a chemical’s concentration in plasma or brain enables simultaneous evaluation of potentially variable pharmacokinetics in differently treated animals. Furthermore, the possibility to quantify the chemicals’ concentrations from very small plasma volumes enables monitoring the pharmacokinetic profiles over time without sacrificing the animals. In addition, multiple repeated analyses or parallel detection of another compound (each requiring *ca*. 50 pL of sample only) can be performed out of the few μL sample volume, if needed.

## Material and Methods

### Reagents

Ammonium sulfate, boric acid, and hydrochloric acid were from Riedel-de Haën (Seelze, Germany). Chloroform, 2-(*N*-Morpholino)ethanesulfonic acid (MES) hydrate, 2-propanol, sodium phosphate dibasic dehydrate, and sodium phosphate monobasic dehydrate were from Sigma-Aldrich (Steinheim, Germany). Sodium hydroxide was from J.T. Baker (Deventer, Netherlands). Morphine hydrochloride was from University Pharmacy (Helsinki, Finland). Water was purified with the Milli-Q water purification system (Millipore, Moslheim, France).

### Plasma and brain samples

The mice were administered morphine hydrochloride (20 mg/kg) intraperitoneally (*i.p*.) by injection through the peritoneal membrane into the abdominal cavity. Thirty minutes afterwards, the mice were sacrificed under deep pentobarbital (200 mg/kg, *i.p*., Mebunat, Orion Pharma, Espoo, Finland) anesthesia. Trunk blood was collected into tubes containing K_3_-EDTA (ethylenediaminetetraacetic acid tripotassium salt) and centrifuged (3000 g, 10 min) at 17 °C. Brains were quickly dissected, frozen on dry ice, and stored at −80 °C. Samples were homogenized mechanically (Ultra-Turrax T8, IKA Labortechnik, Staufen, Germany) in ice-cold water (5 mL in each sample). Before analysis, plasma and brain homogenate samples were treated with off-chip LLE as described below.

All experimental procedures were carried out with the approved permissions (ESAVI-0010026/041003/2010 and ESAVI/3806/04.10.07/2015) and had ethical approval from the State Provincial Government of Southern Finland. All efforts were made to minimize the number and suffering of animals.

### Sample preparation

A 10 mM stock solution of morphine hydrochloride was prepared in water and diluted with the specified buffer solution for method optimization and validation. For method development purposes, blank plasma and brain homogenates from mice not treated with morphine were spiked with known concentrations of morphine.

The LLE method used for mouse plasma samples was adopted with modifications from a previous study^29^. Briefly, 100 µL of saturated ammonium sulfate solution was slowly added to 100 µL of plasma sample. The mixture was centrifuged at 1200 g for 10 min, after which the supernatant was separated from the pellet and mixed with 200 µL of 0.1 M Na_2_HPO_4_ (pH 8.9). Next, the supernatant was extracted with 500 µL of chloroform–isopropanol (9:1) by vortex mixing for 2 min. The organic phase was collected and the extraction step was repeated with another 500 µL of chloroform–isopropanol (9:1). Finally, the organic phase (combined) was evaporated to dryness under nitrogen stream, and the residue was dissolved in 50 µL of 5 mM MES buffer (pH 6.0) upon heating at 37 °C for 30 min, followed by vortex mixing for 30 min.

The brain homogenate samples were also treated by LLE by slowly adding 400 µL of saturated ammonium sulfate solution to 400 µL of brain homogenate. The mixture was centrifuged at 6000 g for 15 min, after which the supernatant was separated from the pellet and mixed with 400 µL of 0.1 M Na_2_HPO_4_ (pH 8.9). The sample was then extracted two times with 1 mL of chloroform–isopropanol (9:1) by vortex mixing for 2 min, followed by evaporation and dissolving steps similar to plasma samples, with the exception that the dry residue was dissolved in 25 µL of buffer instead of 50 µL.

### Microchip design and device details

The hybrid glass/SU-8 microchips (MicruX Technologies, Oviedo, Spain; Fig. 1b) comprised a 35-mm-long separation channel (50 µm × 20 µm, w × h) intersected by a 10-mm-long injection channel (50 µm × 20 µm, w × h; simple cross) and three thin-film platinum electrodes with 100 µm spacing: the working electrode (width 50 µm), the auxiliary electrode (width 250 µm), and the reference electrode (width 250 µm) (Fig. 1c). The effective separation length (L_eff_) between the injection cross and the end-channel electrodes was 30 mm.

### Microchip electrophoresis-electrochemical detection

The microchip was filled and rinsed with the BGE before analysis. MES buffer (20 mM, pH 6.5) was used as the BGE. Samples were diluted in 5 mM MES buffer (pH 6.0) and loaded electrokinetically by applying an electric field between the sample inlet (SI, Fig. 1c) and the sample outlet (SO) for 8–10 s. The electrophoretic separation was performed by applying the electric field between the buffer inlet (BI) and the buffer outlet (BO). Both electrophoretic and electrochemical detection voltages were controlled by the HVStat software provided together with the instrument. The separation conditions were optimized in terms of selectivity and sensitivity, including the BGE composition, the separation voltage, and the working electrode (WE) potential. The robustness of the method was determined in terms of the loading time and the sample volume applied to the sample inlet.

Lower limit of morphine quantification (LLOQ) was preliminarily determined based on precision of morphine quantitation at each concentration between 50 and 500 nM in 5 mM MES buffer (pH 6.0). The method qualification was thereafter carried out by adopting the FDA Guidance for Industry – Bioanalytical Method Validation in relevant parts^31^. Selectivity of the method was demonstrated by comparing the electropherograms of blank plasma (n = 10) and blank brain homogenate samples (n = 6) from mice not treated with morphine to electropherograms of blank matrices spiked with morphine to concentrations of 5 µM (plasma) or 1 µM (brain homogenate). The calibration curve (with 1/Y^2^ weighting) was established between morphine concentrations of 0.5 and 20 µM (in 5 mM MES buffer). Within-run and between-run precision (on three different days) were determined with morphine standards in 5 mM MES buffer (n = 4) at three different concentrations (1, 5, and 20 µM). In addition, within-run precision, accuracy and recovery were determined at three different morphine concentrations (1, 5 and 20 µM, n = 6 each) using spiked blank plasma. The within-run precision, accuracy, and recovery of morphine detection in brain homogenate were also determined using a single morphine concentration of 1 µM (n = 5). The recoveries and preconcentration factors (by LLE) were taken into account in calculation of the unknown morphine concentrations in animal samples.

The raw data related to method optimization, qualification, and analysis of mouse plasma and brain samples are given the Supplementary Dataset file.

## Supplementary information

Dataset 1

## Data Availability

All data generated or analysed during this study are included in this published article and its Supplementary Dataset file.
